# High Density Resistive Array Readout System for Wearable Electronics

**DOI:** 10.3390/s22051878

**Published:** 2022-02-27

**Authors:** Shanthala Lakshminarayana, Younghun Park, Hyusim Park, Sungyong Jung

**Affiliations:** Department of Electrical Engineering, University of Texas at Arlington, Arlington, TX 76019, USA; shanthala.lakshminarayana@mavs.uta.edu (S.L.); younghun.park@mavs.uta.edu (Y.P.)

**Keywords:** wearable, flexible, embedded system, high-density resistive array, Bluetooth Low Energy, point-of-care testing, electronic nose (e-nose), electronic skin (e-skin), wireless sensor network

## Abstract

This work presents a wearable sensing system for high-density resistive array readout. The system comprising readout electronics for a high-density resistive sensor array and a rechargeable battery, was realized in a wristband. The analyzed data with the proposed system can be visualized using a custom graphical user interface (GUI) developed in a personal computer (PC) through a universal serial bus (USB) and using an Android app in smartphones via Bluetooth Low Energy (BLE), respectively. The readout electronics were implemented on a printed circuit board (PCB) and had a compact dimension of 3 cm × 3 cm. It was designed to measure the resistive sensor with a dynamic range of 1 KΩ–1 MΩ and detect a 0.1% change of the base resistance. The system operated at a 5 V supply voltage, and the overall system power consumption was 95 mW. The readout circuit employed a resistance-to-voltage (R-V) conversion topology using a 16-bit analog-to-digital converter (ADC), integrated in the Cypress Programmable System-on-Chip (PSoC^®^) 5LP microcontroller. The device behaves as a universal-type sensing system that can be interfaced with a wide variety of resistive sensors, including chemiresistors, piezoresistors, and thermoelectric sensors, whose resistance variations fall in the target measurement range of 1 KΩ–1 MΩ. The system performance was tested with a 60-resistor array and showed a satisfactory accuracy, with a worst-case error rate up to 2.5%. The developed sensing system shows promising results for applications in the field of the Internet of things (IoT), point-of-care testing (PoCT), and low-cost wearable devices.

## 1. Introduction

Wearable device technologies have been widely used in various head-to-toe applications such as environmental analysis, biomedical, physical, and physiological monitoring, primarily as accessory-type such as gloves, headsets, watches, wristbands, and textiles [1,2,3,4,5]. In current years, wearable systems with unique sensing materials and device structures have proved to be highly sensitive in mimicking the human olfactory system and tracking biophysical and biochemical signals, including skin temperatures, body movements, heart rates, pulse oximetry, blood pressures, breathing, wound healing, as well as continuous biofluid monitoring, for instance, sweat and interstitial fluids. In most cases, accessory-type wearable devices fail to provide an accurate electrode-based physiological detection capability due to unreliable body contact. Therefore, many recent research interests have moved to attachable body devices, including patch and sticker type devices [6,7,8,9,10]. Flexible sensing electrodes are required to implement these attachable devices, and modules should be miniaturized to provide comfortable body wearing. The use of flexible sensor arrays in point-of-care testing (PoCT) has been regarded as a promising approach to monitoring patients out of the hospital and lessening the burden on public healthcare systems in caring for older adults or patients with chronic diseases [11,12].

One of the most popular sensors for a wearable device is a resistive sensor, which has a sensing element whose resistance changes as a function of the target physical or chemical quantity. It is utilized in industrial, scientific, and commercial applications for sensing numerous physical parameters including, but not limited to, ambient temperature, humidity, pressure, strain/force, light intensity, and displacement [13,14,15]. Recent growth in the metal oxide (MOx) semiconductor sensor has enhanced the use of resistive sensors for more sophisticated applications such as chemical sensing, gas sensing, and biosensing [16,17,18,19]. Many resistive sensing materials, such as conductive polymers, nanomaterials that cover single- or multi-wall carbon nanotubes, graphene, and nanoparticles, can be deposited on flexible polyethylene terephthalate (PET) and polyimide-based substrates using inkjet or screen-printing techniques [20,21,22]. An array form of the flexible resistive sensor is developed and adopted in wearable electronic applications, which can help improve the measurement accuracy, sensitivity, and selectivity while helping in multi-analyte detection [11,23,24,25,26,27,28,29,30]. Significant evolutions are engineered in readout electronics for sensing devices parallel to the sensor development. Nowadays, the demand for a modern integrated sensing system is growing that can combine analog or digital sensors, signal conditioning circuits, processing units, and communication interfaces in a single device [31,32,33]. The integrated sensing systems are often equipped with wireless interfaces using a standardized protocol such as Bluetooth Low Energy (BLE), Zigbee, Wi-Fi, LoRa, and Sigfox, enabling them to build wireless sensor networks for the Internet of things (IoT) applications.

Herein, we report the development of a wearable universal-type sensing system comprising miniaturized wireless readout electronics packaged into a wristband form factor. The readout is compatible with any custom or commercial resistive sensor array on a flexible substrate. Based on the type of materials deposited on the sensing array, the system can behave as a universal-type multi-functional sensing system, since it supports different resistive sensors, including chemiresistive, piezoresistive, thermo-electric, and their hybrid. The device can act as a resistive electronic skin (e-skin) for body temperature and blood pressure monitoring, a portable electronic nose (e-nose) for environmental harmful/toxic gas detection, and a comprehensive breathing analyzer. Compared with existing wearable resistive readout devices, the proposed device demonstrated the lowest circuit complexity on a microcontroller-based wearable platform and a high-density sensor array capacity. Thus, it could be an ideal solution for various applications such as wearable wireless sensor networks, PoCT, and battery-powered wireless telemetry for biomedical applications, while presenting a reference for designing low-complexity, low-cost, low-power wearable systems for resistive sensor arrays. The device also supports a wide range of the resistance measurement from 1 KΩ to 1 MΩ, while providing a better tradeoff between the measurement accuracy and the processing speed. The rest of this paper is organized as follows. Section 2 describes the system-level specifications and the architecture of the proposed prototype. Section 3 shows the experimental results and the case analysis with a force-sensitive resistor as proof of concept. Finally, discussions and conclusions are drawn in Section 4.

## 2. System Description and Specification

**Figure 1:**
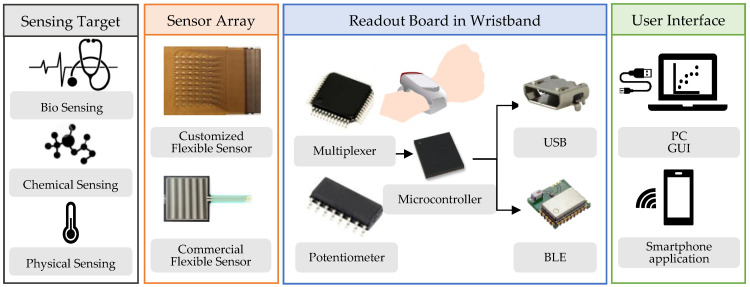
System-level block diagram, conceptually depicting readout electronics housed in a wristband chassis.

The wearable system consists of the following modules: (1) a flexible customized or commercial resistive sensor array to detect target analytes such as biomarkers and chemicals; (2) a readout board to collect, process and transmit sensor data to a personal computer (PC) or a smartphone; and (3) a user-friendly graphical user interface (GUI) to control the device and display the obtained data, as shown in Figure 1. The hardware comprising readout electronics and a battery was housed in a wristband chassis. The system and the sensor array specifications targeted in this work are tabulated in Table 1. The resistance range of the piezoresistive and thermoelectric sensor varied from a few ohms to kiloohms [2,11], whereas the chemiresistive sensor varied up to a few megaohms [23]. Thus, an input resistance range of 1 KΩ–1 MΩ was targeted in this work to cover a variety of resistive sensors.

### 2.1. Readout Board

**Figure 2:**
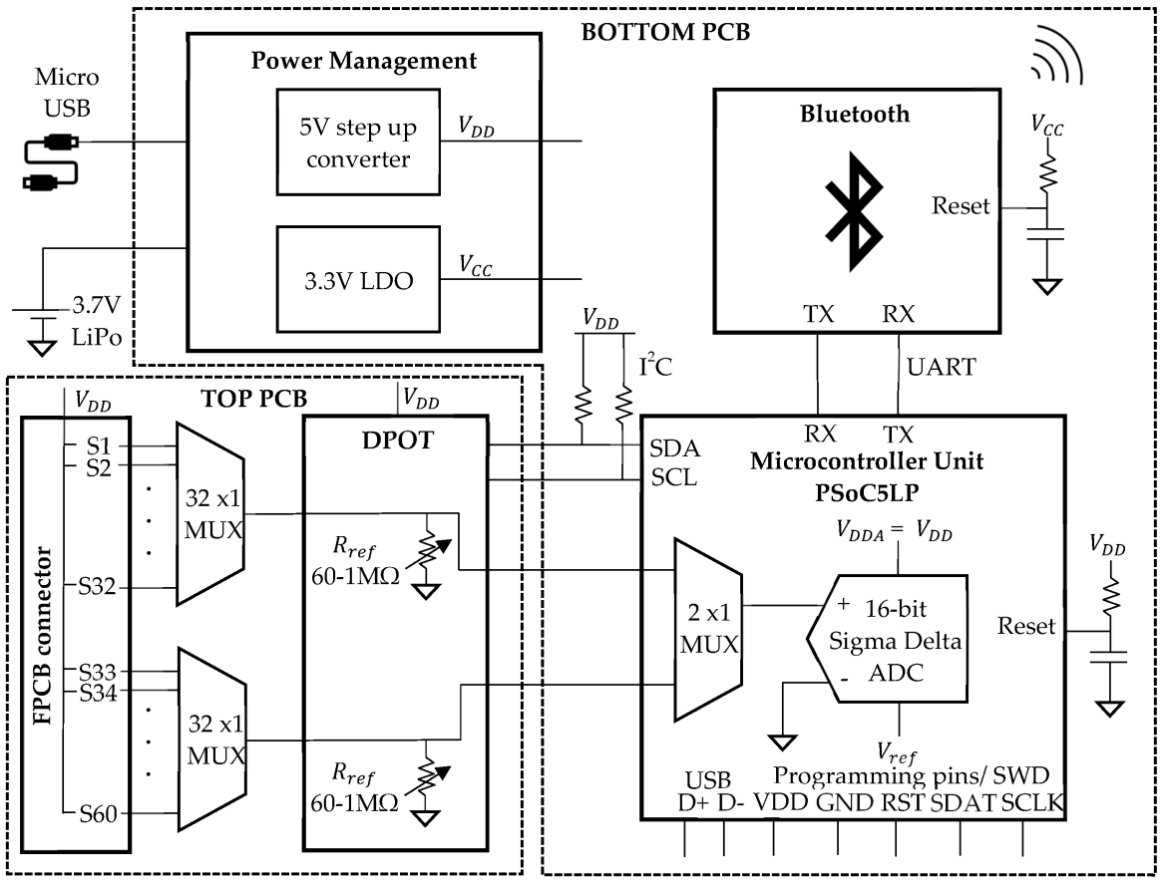
Detailed block diagram of the readout board.

A readout board was required to measure the resistance change of the sensor as a function of the target physical or chemical reaction. A resistance-to-voltage (R–V) conversion was employed in the proposed readout circuit using a voltage divider concept consisting of a target resistive sensor ($R_{sensor}$) and a reference resistor ($R_{ref}$). The function of the readout board involved the following: (1) collecting and multiplexing the sensor output, which was the voltage divider output; (2) processing the collected sensor output and then converting it to the digital form using an analog-to-digital converter (ADC); and (3) sending the converted digitalized data to a PC or smartphone using wired or wireless communication. The subsystems of the readout board were as follows: (1) multiplexers (MUX); (2) a digital potentiometer (DPOT); (3) a microcontroller unit (MCU); (4) a micro-universal serial bus (USB); (5) a Bluetooth unit; and (6) a power management circuit consisting of voltage regulators and a battery management circuit, as illustrated in Figure 2. The readout circuit was fabricated on one top and one bottom printed circuit boards (PCBs). The top PCB contained the MUX and the DPOT, whereas the bottom PCB consisted of the MCU, the BLE unit, and the power management block.

#### 2.1.1. Top PCB Electronics

S1–S60 in Figure 2 represents the 60 two-electrode resistive sensors connected to the readout board via a flexible PCB (FPCB) connector “FH29B-120S-0.2SHW(05)” (Hirose-Connector, Kanagawa, Japan). One electrode of each resistive sensor was connected to the supply voltage ($V_{DD}$) provided by the power management block of the readout board, while the other electrode of each sensor was connected to the multiplexer input. To individually address the 60 different sensors, two 32 × 1 MUX “ADG732BSUZ” (Analog Device, Norwood, MA, USA) were utilized. Due to the broad resistance range of the target sensor (1 KΩ–1 MΩ), using a fixed value of $R_{ref}$ in the voltage divider circuit resulted in a voltage saturation at the ADC input, especially for the sensors that fell in the extreme corners of the target resistance range. To avoid this problem, the $R_{ref}$ value was tuned similar to the $R_{sensor}$ value using a dual-channel 8-bit DPOT “AD5242BRZ1M” (Analog Device, Norwood, USA). The resistance value of the DPOT ($R_{ref}$) can be varied from 60 Ω to 1 MΩ by programming the registers. The DPOT had three terminals: A, B, and wiper. The wiper position of the DPOT was programmed by the Inter-Integrated Circuit (I^2^C) protocol and can be set to 256 distinct positions. Corresponding to the wiper position, the $R_{ref}$ of the readout system can be set and calculated using Equation (1):

(1) $$R_{ref}=R_{WA}(D)=\frac{256}{\begin{array}{c} 256-D \end{array}}\times R_{AB\;}+R_{W},$$

where $R_{WA}$ is the resistance between the DPOT “A” terminal and the wiper terminal, D is the decimal equivalent of the binary code between 0 and 255 which is loaded in the 8-bit register, $R_{AB}$ is the nominal end-to-end resistance and equals to 1 MΩ, and $R_{W}$ is the wiper resistance contributed by the ON-resistance of the internal switch and equals to 60 Ω.

#### 2.1.2. Bottom PCB Electronics

The sensor data collected from the top PCB was sent to a MCU “CY8C5888LTILP097” (Cypress Semiconductors, San Jose, USA), also called PSoC5LP, on the bottom PCB. The PSoC5LP family provides a combination of a 32-bit Arm^®^ Cortex^®^-M3 processor with a flexible, configurable block of an analog subsystem, digital subsystem, routing, and general purpose input/outputs (GPIOs), which enables a high level of integration in a wide variety of applications. The analog multiplexer output was digitalized using an internal PSoC5LP 16-bit ADC operated in the single-ended mode with a rail-to-rail output. Once the digitalized ADC output ($ADC_{voltage}$) was obtained, the unknown value of $R_{sensor}$ can be calculated using Equation (2):

(2) $$R_{sensor}=\frac{R_{ref}\times \left(V_{DD}-ADC_{voltage}\right)}{\begin{array}{c} ADC_{voltage} \end{array}}.$$

where $R_{sensor}$ is the unknown sensor resistance, $R_{ref}$ is the reference DPOT resistance, $V_{DD}$ is the supply voltage, and $ADC_{voltage}$ is the digitalized ADC output. The ADC step corresponded to 76 µV, which could detect a 0.1% variation of the sensor output with a resistance range between 1 KΩ and 1 MΩ (i.e., 1 Ω change for the 1 KΩ base sensor resistance and 1 KΩ change for the 1 MΩ base sensor resistance). The readout board was designed to operate at a $V_{DD}$ of 5 V. The power could be provided to the board by using a USB cable or a rechargeable battery. A 3.7 V and 500 mAh lithium-ion polymer battery (LiPo) was employed in the prototype, which fit the 3 cm × 3 cm wristband chassis. To provide a constant 5 V supply, a step-up/boost converter “TPS61240IDRVRQ1” (Texas Instruments, Dallas, USA) was adopted. A low-dropout (LDO) voltage regulator “TLV75733PDBVR” (Texas Instruments, Dallas, USA) was selected to provide a constant voltage $V_{CC}$ of 3.3 V to the BLE technology. The processed information was transferred to the user’s smartphone via a BLE unit “SPBTLE-1S” (STMicroelectronics, Geneva, Switzerland). The BLE unit was interfaced with the MCU through the Universal Asynchronous Receiver/Transmitter (UART) protocol at a baud rate of 115,200 bits per second. The board also included a type B micro-USB “UJ2-MIBH2-4-SMT-TR” (CUI device, Lake Oswego, OR, USA), which powered the system and communicated with a PC using USB 2.0 standards. The detailed specifications of the components considered during the design are listed in Table 2.

**Figure 3:**
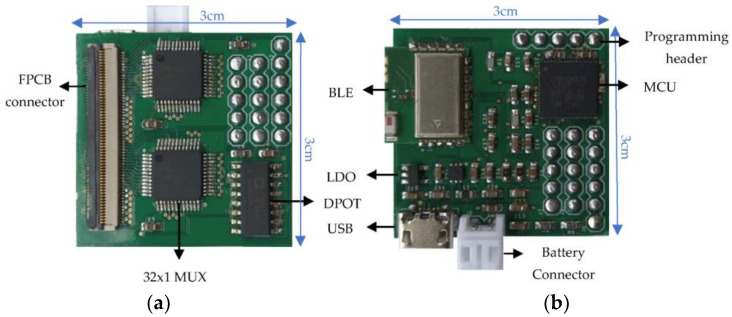
(**a**) Assembled top printed circuit board (PCB) with labelled parts (3 cm × 3 cm × 0.2 mm); (**b**) assembled bottom PCB with labelled parts (3 cm × 3 cm × 0.2 mm).

The photos of the assembled top and bottom PCBs are shown in Figure 3. The small form factor is an essential criterion to make the device suitable for wearable applications. To obtain a small form factor, two 0.2 mm thick PCBs were designed to stack them back-to-back, instead of designing the readout circuit on a single large PCB. Two two-layered PCBs were preferred as a substitute for a four-layered PCB to reduce the fabrication cost. Both the dimensions of the top and bottom PCBs were 3 cm × 3 cm, making the device suitable for wearing as a wristband. The “Eagle 9.4.2” software (Autodesk, San Rafael, CA, USA) was used to design the layout of the two-layer readout PCBs.

### 2.2. Firmware and Algorithm

**Figure 4:**
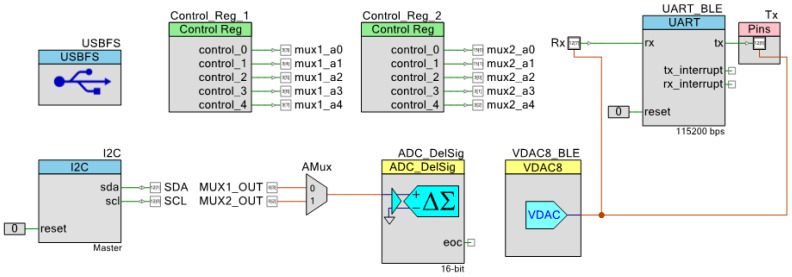
System firmware architecture in the programmable system-on-chip (PSoC) creator.

To develop the firmware for the readout board, the following subsystems of PSoC5LP were utilized, as shown in Figure 4:

- An I^2^C block to communicate with the DPOT;
- A 16-bit Delta-Sigma ADC;
- Control registers to control the selection bits of 32×1 MUX;
- 2 × 1 analog MUX to combine the two 32 × 1 MUX outputs;
- A USB block to control the USB2.0 bus;
- A UART block to communicate with the BLE unit;
- A voltage digital-to-analog converter (VDAC) block to adjust the voltage of signals for UART transmit (TX) and receive (RX) lines.

The source code was written in the embedded C programming language and was compiled using a “PSoC Creator 4.2” compiler released by Cypress semiconductors. A “CY8CKIT-002 PSoC MiniProg3 Program & Debug Kit” (Cypress Semiconductor, San Jose, USA) was utilized to test and debug the board functionalities through Serial Wire Debug (SWD).

**Figure 5:**
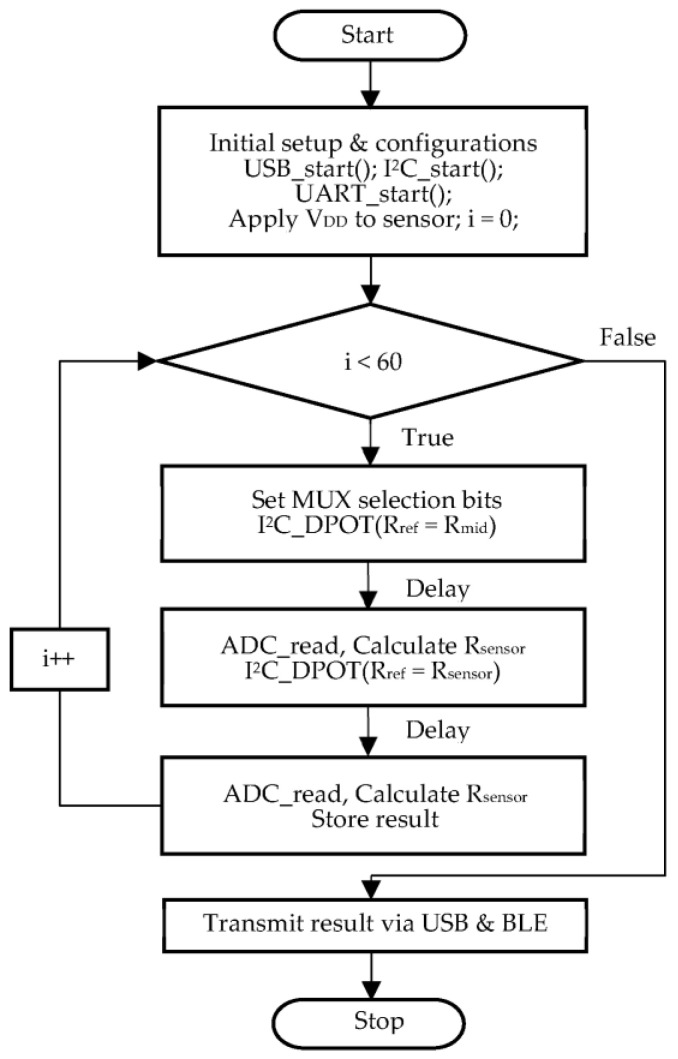
Flowchart of the proposed system.

The flow chart of the proposed system is presented in Figure 5. After the system initialization was completed, a supply voltage was applied to the sensors. The finalized $R_{sensor}\;$was obtained after tuning the $R_{ref}$ to match the value of $R_{sensor}$. A few steps were required in the firmware to achieve tuned $R_{ref}$. First, the value of $R_{ref}\;$was set to mid-range ($R_{mid}$), and $ADC_{voltage}$ was obtained. Using the $ADC_{voltage}$ and Equation (2), the value of $R_{sensor}\;$was calculated. During the next step, the value of $R_{ref}$ was set to the computed value of $R_{sensor}\;$in the previous step. Thus, the process automatically balanced the value of $R_{ref}$ to $R_{sensor}\;$and avoid the voltage saturation problem caused by the fixed $R_{ref}$, which resulted in accurate $R_{sensor}$ values. The 60 sensors were measured one after the other in series, and equating $R_{ref}$ to $R_{sensor}$ was performed every time before reading each sensor, using the firmware coding. Thus, the process was repeated 60 times until all the sensor values were calculated, and the obtained sensor data ($R_{sensor}$) were transferred to a PC via USB or a smartphone via BLE.

### 2.3. User Interface: GUI and Smartphone Application

A customized GUI was developed to connect the readout board and the PC using a USB Communication Port (COM port). The GUI written in Python provided a real-time display plot to visualize the captured sensor data from the device and saved the obtained sensor data in a Comma Separated Values (CSV) format file. The “Tkinter” GUI framework and the “matplotlib” library were utilized to build the mentioned functionalities. Meanwhile, the USB communication was established using the “pySerial” library, and all the libraries were compiled into a single executable file using the “pyinstaller” library.

**Figure 6:**
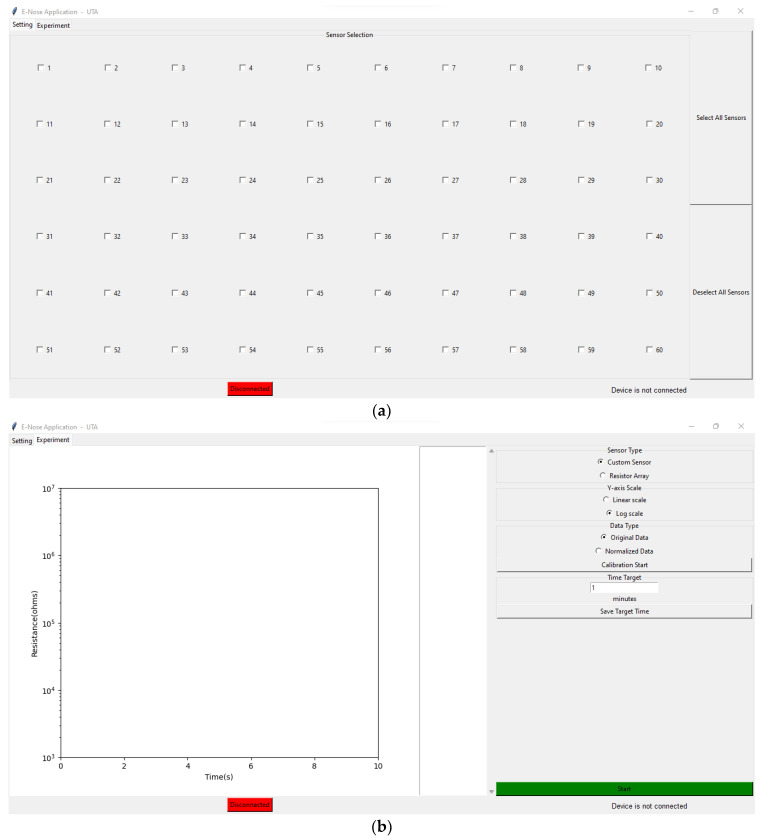
(**a**) Sensor setting window of the graphical user interface (GUI); (**b**) experiment window of the GUI.

Figure 6 illustrates the sensor setting window and the experiment window of the customized GUI. The sensor setting window of the GUI consisted of 60 checkboxes to individually select/unselect a sensor and a connectivity check button. If the device was connected successfully, the button would turn green and showed “Connected”; otherwise, it showed “Disconnected” in red. The experiment window allowed the user to set the target experiment duration, perform sensor calibration, visualize the resistor values in a graph, select the *X*-axis to linear or log scale and start/stop the experiment.

Meantime, the user can also use a smartphone application as a user interface to receive the data from the readout board. The “BLE Scanner” application (Bluepixel Technologies LLP, Ahmedabad, India) was used as a prototype to display the raw resistance values of the sensors, which were transmitted by the BLE unit in the board. The SPBTLE-1S BLE module was compliant with Bluetooth^®^ specifications v4.2 with an embedded ceramic antenna that operated at 2.4 GHz. The device was recognizable with the name “BlueNRG-1” in the “BLE Scanner” application to connect with the readout board. Once the connection was made, communication could occur between the readout board and the smartphone through BLE.

## 3. Results

### 3.1. Case Analysis 1: Test Setup with a Discrete Resistor Array and Electrical Testing Results

**Figure 7:**
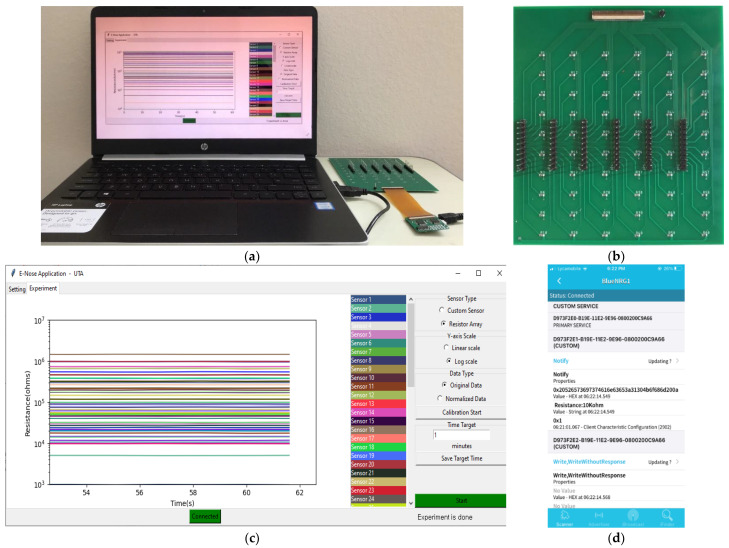
(**a**) Test setup with the readout board and the 60-resistor array; (**b**) 60-resistor array; (**c**) 60 resistor results displayed in the GUI; (**d**) system response displayed in a smartphone.

The proposed system was tested electrically for verification, and Figure 7a illustrates the test setup. To mimic the 60-sensor array, an electrical equivalent model with a 60-resistor array was fabricated on a PCB, as shown in Figure 7b. The 60-resistor array was realized by 0.1%-tolerance thin-film discrete resistors, where the resistance varied from 1 KΩ to 1 MΩ. The resistor array was connected to the readout board using an FPCB jumper cable. The USB cable connected the readout board and the customized PC GUI to power up the board. In the GUI, the resistance values of 60 sensors were displayed (Figure 7c), which varied with respect to time, and the results were saved in the CSV format file to record the sensor response. Figure 7d shows the system response displayed in the smartphone.

**Figure 8:**
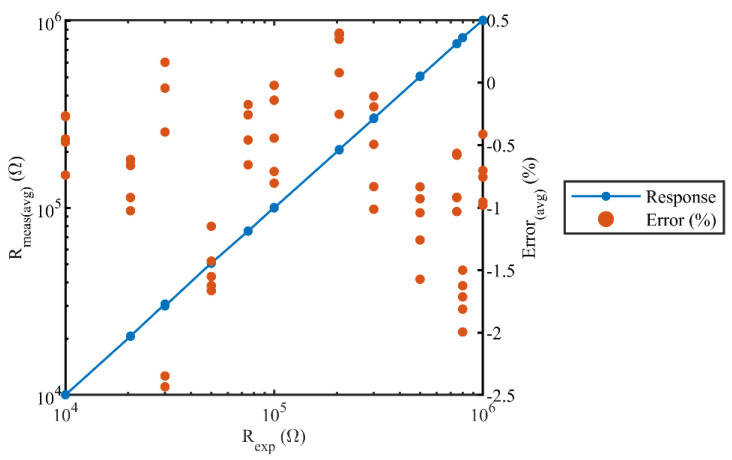
System response depicting the expected versus measured resistances and the average errors.

**Figure 9:**
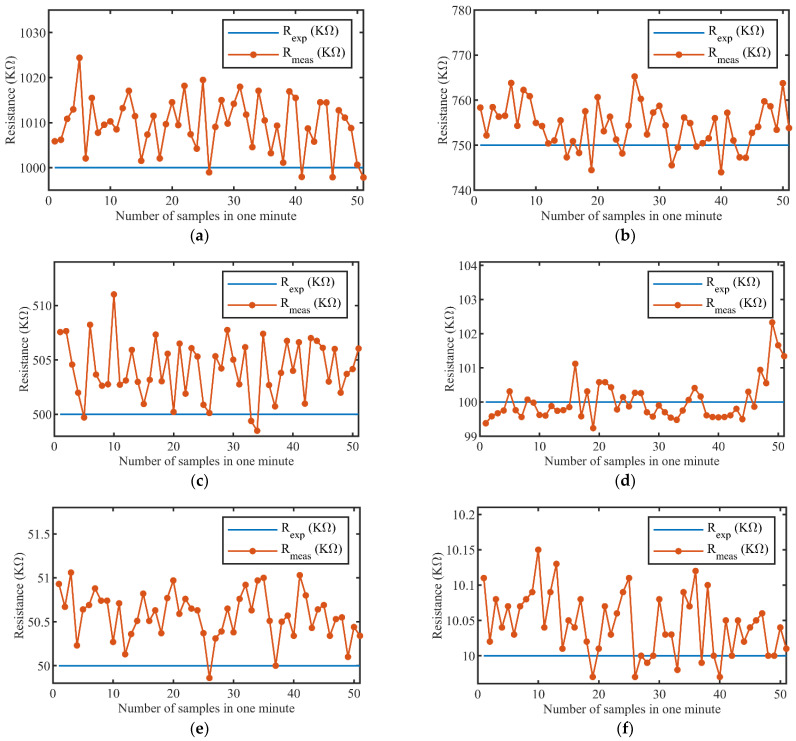
System’s transient response: (**a**) $R_{exp}$= 1 MΩ; (**b**) $R_{exp}$ = 750 KΩ; (**c**) $R_{exp}$ = 500 KΩ; (**d**) $R_{exp}$ = 100 KΩ; (**e**) $R_{exp}$ = 50 KΩ; (**f**) $R_{exp}$ = 10 KΩ.

To validate the error rate of the proposed system, Equations (3)–(5) were utilized:

(3) $$Error_{\left(avg\right)}{\left(\%\right)}_{\;}=\frac{\;R_{exp}-R_{meas\left(avg\right)}\;}{\begin{array}{c} R_{exp} \end{array}}\times 100,$$

(4) $$Error_{\left(max\right)}{\left(\%\right)}_{\;}=\frac{\;R_{exp}-R_{meas\left(max\right)}\;}{\begin{array}{c} R_{exp} \end{array}}\times 100,$$

(5) $$Error_{\left(min\right)}{\left(\%\right)}_{\;}=\frac{\;R_{exp}-R_{meas\left(min\right)}\;}{\begin{array}{c} R_{exp} \end{array}}\times 100,$$

where $R_{exp}$ is the expected sensor resistance value measured using a digital multimeter “Agilent 34401A” (Keysight Technologies, Santa Rosa, USA) and $R_{meas}$ is the measured sensor resistance value obtained using the proposed readout board. In a span of 60 s (1 min), 60 sensor data were collected 51 times, resulting in 3060 sensor samples. These samples were used to calculate the error rates as per Equations (3)–(5). The maximum, minimum, and average values of the measured sensors resistances and the corresponding values of error rates are listed in Table 3. The sensor response $R_{exp}$ versus $R_{meas\left(avg\right)}\;$showed a linear trend over the target measurement range, as depicted in Figure 8. The worst-case error rate reported during the experiment was under 2.5%. Since it was tedious to display the system’s transient response for all 60 values of the sensors, the transient response is illustrated for a span of one minute in Figure 9, with only six different sensor resistances, which were 1 MΩ, 750 KΩ, 500 KΩ, 100 KΩ, 50 KΩ, and 10 KΩ.

### 3.2. Case Analysis 2: Test Setup with a Force-Sensitive Resistor and Sensor Testing Results

**Figure 10:**
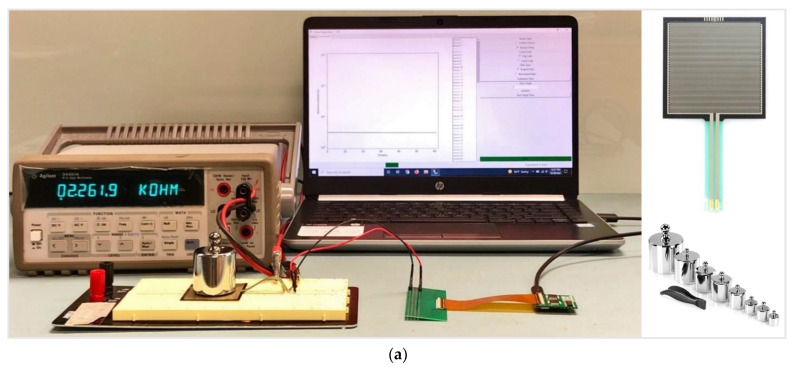

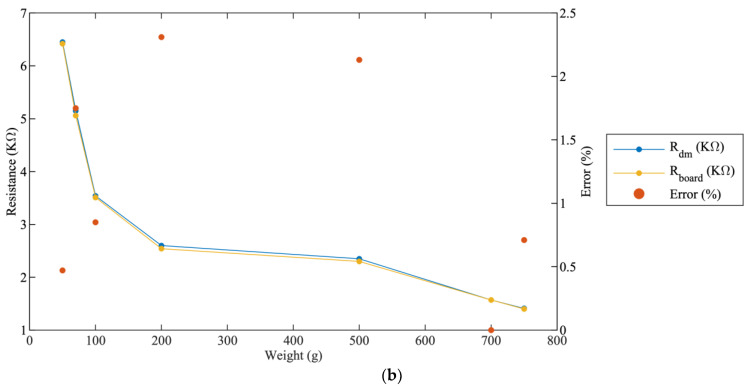
(**a**) Test setup with the readout board, “FSR-406”, and weights; (**b**) comparison of the “FSR-406” responses using a digital multimeter and the readout board.

As the second case analysis, the developed system was tested by interfacing with a commercially available force-sensitive resistor “FSR-406” (Interlink Electronics^®^, Camarillo, USA). The “FSR-406” varies its resistance, depending on the force applied to the sensing area. The non-actuated sensor resistance is larger than 1 MΩ, and its resistance decreases exponentially as per the force applied on the sensing area. The sensor had a sensing area of 0.75” × 1.5” and can sense applied force in the range of 10 g to 10 Kg. During the experiment, “FSR-406” was connected to the readout board, as illustrated in Figure 10a, and the sensor response measured by the readout board ($R_{board}$) was recorded throughout the applied weight range from 50 g to 750 g. Simultaneously, the sensor response measured by a digital multimeter ($R_{dm}$) was also captured using the digital multimeter “Agilent 34401A” (Keysight Technologies, Santa Rosa, USA). Ten readings were taken and averaged to obtain both $R_{board}\;\mathrm{and}\;R_{dm}$. The comparison results between $R_{board}$, $R_{dm}$, and the corresponding error rates calculated during the experiment are listed in Table 4. The response of the designed readout board showed a close correlation with the response measured using a digital multimeter, demonstrating a satisfactory performance as illustrated in Figure 10b. Due to the unstable response of “FSR-406” at lower weights, the experiments were conducted for weights of 50 g and above to avoid inaccurate results.

## 4. Discussion and Conclusions

The overall system performance is summarized in Table 5. In a span of 60 s (1 min), 60 sensor readings were collected and stored in the CSV file 51 times, resulting in a total of 3060 sensor measurements. This led to a processing speed of 51 Hz, equivalent to 19.6 ms, to collect, process and transmit the data from a single sensor to the GUI. The processing speed often depends on the input resistance range. Thus, the system targeting solely piezoresistive sensors provided a somewhat faster response due to the lower target resistance. However, typical e-nose gas detection systems, which target chemiresistive sensors of a wide range up to 1 MΩ, offer a processing speed of 0.1 Hz to 10 Hz (10 s to 100 ms per sensor) [33]. Compared with those systems, the proposed method provides a processing speed of 51 Hz (19.6 ms per sensor), which is significantly faster. Also, an application such as a comprehensive breathing analyzer requires a sampling frequency range of 0.1 Hz to 0.8 Hz [5], which can be easily satisfied by the proposed system. The readout system achieved a worst-case error rate of less than 2.5%. These variations were caused by the combination of the supply voltage fluctuation of regulators by ±2%, the DPOT error rate of ±0.5 least significant bit (LSB), the 0.1% tolerance of thin-film discrete resistors in the resistor array, and the ADC conversion error. The system power consumption was around 95 mW, and the majority of the power was consumed while the BLE was transmitting the data to the user. The low-power-consumption profile makes the device suitable for battery-powered wireless sensor networks.

The possible limitation of the system can be its scalability to adopt a more extensive size array. The expansion of the system to more than 60 sensors makes the system bulkier and might not be appropriate for wearable electronics. As future work, the prototype will be tested chemically by interfacing with chemiresistive gas sensors to detect the target analytes of various concentrations.

To summarize, a compact universal-type resistive sensor array readout based on the R–V conversion is presented. This work demonstrates the novelty and potential application of an integrated wearable sensing system with the following attributes: a low-cost system with a wireless communication capability, a facility to accommodate a high-density resistive sensor array, and portable readout electronics with a small form factor of 3 cm × 3 cm. The system behaves as an electrophysiological sensing interface for wearable healthcare monitoring systems, supporting multi-functionality hybrid sensing, and can be implemented as a readout for e-skin or e-nose. These advantages, especially low-cost, small size, and low power consumption, allow the device to be utilized in battery-powered wireless sensor network applications and next-generation self-sustainable integrated wearable systems in the IoT era.

## Figures and Tables

**Table 1:** System specifications.

Sensor Type	Resistive sensor
Sensor resistance range	1 KΩ–1 MΩ
Number of sensors	60; can be expandable
Sensor sensitivity	0.1%
Target processing time	~1 s
Target applications	Electronic skin (e-skin) for blood pressure and body temperature monitoring; electronic nose (e-nose) for environmental harmful/toxic gas detection

**Table 2:** Component specifications considered for the top and bottom PCBs designs.

Parameter	Value	Unit
ADG732BSUZ specifications		
Operating supply voltage	1.8–5.5	V
Supply current	20	µA
ON-resistance	5.5	Ω
AD5242BRZ1M specifications		
Operating supply voltage	2.7–5.5	V
Supply current	100	nA
Digital potentiometer (DPOT) resistance range	60–1 M	Ω
PSoC5LP specifications		
Operating supply voltage	1.71–5.5	V
Supply current at 6 MHz	3.1	mA
Supply current at 24 MHz	8.9	mA
Supply current in the sleep mode	2	µA
Internal analog-to-digital converter (ADC) input range	0–5	V
Internal ADC reference voltage (V*_ref_* = V*_DDA_*/4)	1.25	V
Internal ADC conversion rate	10	KSPS
TPS61240IDRVRQ1 specifications		
Output voltage	5 ± 2%	V
Input voltage range	2.3–5.5	V
Supply current	30	µA
Output current	450	mA
TLV75733PDBVR specifications		
Output voltage	3.3 ± 1%	V
Input voltage range	1.45–5.5	V
Supply current	25	µA
Output current	1	A
SPBTLE-1S specifications		
Operating supply voltage	1.7–3.6	V
Bluetooth version	v4.2	NA
Radiated transmit power	+4	dBm
Receiver sensitivity	−84	dBm
Antenna frequency	2402–2480	MHz
Supply current while receiving	7.7	mA
Supply current while transmitting at 5 dBm	15	mA
Supply current while transmitting at 0 dBm	11	mA
Supply current while in the sleep mode	0.9	µA

**Table 3:** Sixty sensor readings—expected versus measured sensor resistances.

Sensor	$R_{exp}\;(Ω)$	$R_{meas\left(avg\right)}\;(Ω)$	$R_{meas\left(max\right)}\;(Ω)$	$R_{meas\left(min\right)}\;(Ω)$	$Error_{\left(avg\right)}\;(\%)$	$Error_{\left(max\right)}\;(\%)$	$Error_{\left(min\right)}\;(\%)$
R1–R5	1,000,000	1,009,537.00	1,024,401.20	997,827.65	−0.95	−2.44	0.22
R6–R10	800,000	811,997.40	818,773.68	800,976.85	−1.50	−2.35	−0.12
R11–R15	750,000	754,240.60	765,247.17	743,990.80	−0.57	−2.03	0.80
R16–R20	500,000	504,167.20	511,015.70	498,515.00	−0.83	−2.20	0.30
R21–R25	300,000	301,481.70	305,967.77	298,185.59	−0.49	−1.99	0.60
R26–R30	205,000	205,518.40	206,714.55	200,183.09	−0.25	−0.84	2.35
R31–R35	100,000	100,021.60	102,327.48	99,236.38	−0.02	−2.33	0.76
R36–R40	75,000	75,192.49	75,826.99	74,634.44	−0.26	−1.10	0.49
R41–R45	50,000	50,574.35	51,057.00	49,858.35	−1.15	−2.11	0.28
R46–R50	30,000	30,729.80	30,745.39	29,690.22	−2.43	−2.48	1.03
R51–R55	20,500	20,627.91	20,882.63	20,258.24	−0.62	−1.87	1.18
R56–R60	10,000	10,045.01	10,149.11	9971.02	−0.45	−1.49	0.29

**Table 4:** Results of the readout board with “FSR-406” for the weight range of 50–750 g.

Weight (g)	$R_{dm}\;(KΩ)$	$R_{board}\;(KΩ)$	*Error* (%)
50	6.45	6.42	0.47
70	5.15	5.06	1.75
100	3.54	3.51	0.85
200	2.60	2.54	2.31
500	2.35	2.30	2.13
700	1.57	1.57	0.00
750	1.41	1.40	0.71

**Table 5:** System performance summary.

Measurable resistance range	1 KΩ–1 MΩ
Error rate	0–2.5%
Number of sensors	60; can be expandable
Device dimension	3 cm × 3 cm
Supply voltage	5 V
System power consumption	~95 mW at 5 V
Processing speed/data rate	51 Hz
ADC resolution	16 bits
Approximate system cost	$100

## Data Availability

The datasets generated from the current study are available from the corresponding author on reasonable request.
